# Plerixafor (a CXCR4 antagonist) following myeloablative allogeneic hematopoietic stem cell transplantation enhances hematopoietic recovery

**DOI:** 10.1186/s13045-016-0301-2

**Published:** 2016-08-17

**Authors:** Michael M. B. Green, Nelson Chao, Saurabh Chhabra, Kelly Corbet, Cristina Gasparetto, Ari Horwitz, Zhiguo Li, Jagadish Kummetha Venkata, Gwynn Long, Alice Mims, David Rizzieri, Stefanie Sarantopoulos, Robert Stuart, Anthony D. Sung, Keith M. Sullivan, Luciano Costa, Mitchell Horwitz, Yubin Kang

**Affiliations:** 1 Division of Hematologic Malignancies and Cellular Therapy, Duke Cancer Institute, Duke University Medical Center, Durham, NC USA; 2 Division of Hematology/Oncology, Hollings Cancer Center, Medical University of South Carolina, Charleston, SC USA; 3 Duke University Department of Biostatistics and Bioinformatics, Durham, NC USA; 4 Division of Hematology, Department of Medicine, The Ohio State University, Columbus, OH USA; 5 Division of Hematological Malignancies and Cellular Therapy, Duke University Medical Center, Box 3961, 2400 Pratt Street, Durham, NC 27710 USA

**Keywords:** CXCR4, Antagonist, Stromal-derived factor-1, Hematopoietic stem cell transplantation, Hematopoietic stem cells, Outcomes, Neutrophil engraftment, Platelet engraftment

## Abstract

**Background:**

The binding of CXCR4 with its ligand (stromal-derived factor-1) maintains hematopoietic stem/progenitor cells (HSPCs) in a quiescent state. We hypothesized that blocking CXCR4/SDF-1 interaction after hematopoietic stem cell transplantation (HSCT) promotes hematopoiesis by inducing HSC proliferation.

**Methods:**

We conducted a phase I/II trial of plerixafor on hematopoietic cell recovery following myeloablative allogeneic HSCT. Patients with hematologic malignancies receiving myeloablative conditioning were enrolled. Plerixafor 240 μg/kg was administered subcutaneously every other day beginning day +2 until day +21 or until neutrophil recovery. The primary efficacy endpoints of the study were time to absolute neutrophil count >500/μl and platelet count >20,000/μl. The cumulative incidence of neutrophil and platelet engraftment of the study cohort was compared to that of a cohort of 95 allogeneic peripheral blood stem cell transplant recipients treated during the same period of time and who received similar conditioning and graft-versus-host disease prophylaxis.

**Results:**

Thirty patients received plerixafor following peripheral blood stem cell (*n* = 28) (PBSC) or bone marrow (*n* = 2) transplantation. Adverse events attributable to plerixafor were mild and indistinguishable from effects of conditioning. The kinetics of neutrophil and platelet engraftment, as demonstrated by cumulative incidence, from the 28 study subjects receiving PBSC showed faster neutrophil (*p* = 0.04) and platelet recovery >20 K (*p* = 0.04) compared to the controls.

**Conclusions:**

Our study demonstrated that plerixafor can be given safely following myeloablative HSCT. It provides proof of principle that blocking CXCR4 after HSCT enhances hematopoietic recovery. Larger, confirmatory studies in other settings are warranted.

**Trial registration:**

ClinicalTrials.gov NCT01280955

## Background

Despite its curative potential, myeloablative allogeneic hematopoietic stem cell transplantation (HSCT) is associated with a high incidence of morbidity and mortality [1, 2]. Approximately 25 % of patients will die within 1 year of transplant-related complications. Many of these complications arise during the period of bone marrow aplasia that precedes donor cell engraftment. Infection is the most common cause of morbidity and mortality immediately following HSCT. The speed of hematopoietic recovery and donor cell engraftment has been correlated with the length of the hospital stay, the severity of illness during the stay, incidence of infection, and the patient’s survival [3]. Reduction in the period of bone marrow aplasia would be expected to reduce transplant-related mortality. Hence, there is urgent need to develop novel approaches for enhancing hematological recovery in HSCT.

The interaction between stromal cell-derived factor 1α (SDF-1, also called CXCL12) and the CXCR4 chemokine receptor plays a critical role in homing of hematopoietic stem cells to the bone marrow niche. SDF-1 is secreted by endothelial cells, osteoblasts, and other stromal cells and is present in the bone marrow (BM) extracellular matrix. CXCR4 is expressed on hematopoietic stem/ progenitor cells (HSCs) and many other cells. The interaction between SDF-1 in the BM extracellular matrix and the CXCR4 receptors on HSCs has dual effects: tethering HSCs in the niche and arresting the cycling of very primitive HSCs [4–6]. By keeping HSCs in a quiescent state, this proliferation inhibitory effect of CXCR4/SDF-1 interaction may delay the reconstitution and recovery of donor HSCs in the early phase following HSCT.

We thus hypothesized that blocking CXCR4/SDF-1 interaction after transplant would remove this inhibitory effect of the CXCR4/SDF-1 interaction on HSCs, resulting in enhance donor cell reconstitution following HSCT. Additionally, we reasoned that because CXCR4 is expressed on T cells, dendritic cells, and many other cells that produce inflammatory cytokines, CXCR4/SDF-1 blockade would attenuate transplant-related cytokine storm. The massive cytokine storm from conditioning has deleterious effects on hematopoiesis [7–10]. Consistent with these hypotheses, we demonstrated in our preclinical murine study that administration of AMD3100 (an CXCR4 antagonist, also called plerixafor or Mozobil® Sanofi USA) following myeloablative stem cell transplantation resulted in improved animal survival, reduced pro-inflammatory cytokine/chemokine production, and enhanced recovery of all donor cell lineages (myeloid, T and B lymphocytes, erythrocytes, and platelets) [11]. Here, we report our phase I/II study aimed at determining the safety and the efficacy of post-transplant administration of plerixafor in patients receiving myeloablative allogeneic HSCT.

## Methods

### Patients

Patients with high-risk hematologic diseases (acute lymphoblastic leukemia, acute myeloid leukemia, chronic myeloid leukemia, high-grade B cell lymphomas, Hodgkin’s lymphoma, myelodysplastic syndrome, and myelofibrosis) and candidates for myeloablative allogeneic HSCT were eligible for participation. Patients were 18 years of age or older and had an human leukocyte antigen (HLA)-matched sibling or HLA-matched unrelated donor. The trial was approved by the Duke University and Medical University of South Carolina Institutional Review Board and was carried out in accordance with the Helsinki Principle. The clinical trial was registered at www.clinicaltrials.gov as NCT01280955. Informed consent was obtained from all participants.

A cohort of 95 patients from the Duke HSCT program was identified for comparison during analysis. These patients received transplantation concurrently with the study group, as to control for supportive care and standard management practices of the time. Although eligible for the study, these patients were not enrolled for various reasons, including declining participation in the clinical trial, lack of insurance approval for participation, or managing physician preference. This control group had comparable diseases, underwent similar conditioning regimens, and received the identical graft-versus-host disease prophylaxis. Hematopoietic growth factor support was not routinely administered in either group.

### Study design

**Fig. 1:**
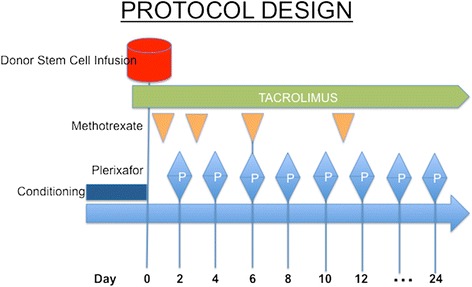
Scheme of the clinical study

The study was a two-stage, phase 1–2, open-label, two-center trial. Patients were conditioned with a myeloablative regimen such as total body irradiation (TBI) (1350 cGy)/cyclophosphamide, TBI/VP-16, busulfan/cyclophosphamide, or busulfan/fludarabine (Table 1). The donor stem cell grafts were from either granulocyte colony-stimulating factor (G-CSF) mobilized peripheral blood stem cells or BM from 8/8 or 7/8 HLA-identical family members or 8/8 (HLA A, B, C, DR*β*1) allele-level matched unrelated donors. The target CD34+ cell dose for mobilized peripheral blood stem cell and BM stem cell recipients was 5 × 10^6^/kg and 2 × 10^6^/kg recipient ideal body weight. Prophylaxis for acute graft-versus-host disease was provided with tacrolimus and methotrexate in all patients. After donor cell infusions, patients received plerixafor given subcutaneously at 240 μg/kg every other day beginning at day +2 after transplant until day +21 or neutrophil engraftment, whichever came first. Phase I was a standard 3 + 3 design to evaluate safety. Three patients were enrolled in phase I portion of the study, and after all three patients demonstrated no dose-limiting toxicities, the study proceeded to phase II. The phase II portion involved the same study schema (Fig. 1). A subset of patients had a BM aspirate and biopsy at approximately day +30 after HSCT.

**Table 1:** Patient and transplant characteristics

Characteristics	Plerixafor group	Control group	*p* value
	*n* = 30	*n* = 95	
Age (years), median (range)	47 (20–61)	50 (19–67)	0.06
Sex			0.89
Male	15 (50 %)	51 (54 %)	
Female	15 (50 %)	44 (46 %)	
Diagnoses			0.13
ALL	11 (36 %)	10 (11 %)	
AML/MDS	15 (50 %)	60 (63 %)	
Chronic lymphocytic leukemia		3 (3 %)	
Chronic myelogenous leukemia	2 (6 %)	5 (5 %)	
Hodgkin’s lymphoma		1 (1 %)	
Multiple myeloma		1 (7 %)	
Mycosis fungoides		1 (1 %)	
Myelofibrosis	1 (3 %)	3 (3 %)	
Myeloproliferative disorder		3 (3 %)	
Non-Hodgkin’s lymphoma	1 (3 %)	7 (7 %)	
Conditioning regimen			0.23
TBI + cyclophosphamide	12 (40 %)	20 (21 %)	
TBI + fludarabine		1 (1 %)	
TBI + melphalan		1 (1 %)	
TBI + VP-16	1 (3 %)	7 (7 %)	
Busulfan + cyclophosphamide	13 (43 %)	34 (35 %)	
Busulfan + fludarabine	4 (13 %)	31 (32 %)	
Fludarabine + melphalan		1 (1 %)	
GVHD prophylaxis			
Tacrolimus + methotrexate	30 (100 %)	95 (100 %)	
Donor type			0.85
Matched related	11 (37 %)	31 (33 %)	
Matched unrelated	19 (63 %)	64 (67 %)	
Graft source			0.09
Bone marrow	2 (6 %)	0	
Peripheral blood stem cells	28 (94 %)	95 (100 %)	
Total CD34+ cell dose (×10^^6^ cells/kg) median (range)	6.01 (1.25–8.5)	6.93 (1.49–10.3)	0.09

*Note*: Comparisons among the two groups were performed by the use of the extended Fisher exact test for categorical variables and the Kruskal-Wallis test for continuous variables

### Endpoints and assessments

The primary endpoint was safety determined according to frequencies and severities of adverse events and was assessed daily during the study. Duke Cancer Institute safety oversight committee evaluated all serious adverse events and events leading to patient withdrawal from the study. Other primary objectives of the trial were to determine plerixafor-associated adverse events, time to neutrophil recovery, and time to platelet recovery. Toxicities were graded using CTCAE version 4.0.3. Neutrophil engraftment was defined as absolute neutrophil count (ANC) of ≥0.5 × 10^9^/L (500/mm^3^) for three consecutive laboratory values obtained on different days with ANC recovery being the first of the three consecutive days. Platelet engraftment was defined as platelet count ≥20 × 10^9^/L for three consecutive days without a platelet transfusion for the preceding 7 days with platelet recovery being the first of the three consecutive days.

The secondary objectives were the incidence of grade II–IV acute graft-versus-host disease graded per NIH consensus criteria, overall survival at day +100, immunological reconstitution as determined by CD4 count, CD8 count, NK cells, and B cell pre-transplant, day +30, +60, and +90, and correlative laboratory studies assessing plasma cytokines/chemokines (IL-12, IFN-λ, TNF-α, and macrophage inhibitory protein-1) measured at day +7, +14, and +30.

### Immune reconstitution

Quantification of the following subsets was performed by flow cytometry on fresh peripheral blood before transplantation and the 30, 60, and 90 days after transplantation: B cells (CD19+, CD3−,CD16−,CD56−), natural killer (NK) (CD3−,CD16+/CD56+) and natural killer T (NKT) (CD3+,CD16+/CD56+) cells, CD3+,CD4+,CD8+, regulatory (CD4+, CD25+, CD62L+), plasmacytoid dendritic cells (DCs) (CD123+, CD11c−), and myeloid DCs (CD123−,CD11c+).

### Cytokine assay

Blood and BM samples obtained at the indicated time points were assayed for IL-12, IFN-λ, TNF-α, and macrophage inhibitory protein-1β (MIP-1β) using ELISA. The coating antibodies and the capturing antibodies for IL-12, IFN-λ, TNF-α, and macrophage inhibitory protein-1 (MIP-1β) as well as the standards were purchased from Novex™ by Life Technologies, and the ELISA was performed as per the manufacture’s recommendation.

### Statistical analysis

All patient characteristics were compared using the Student’s *t* test for continuous variables and the chi-square test for categorical variables. The cumulative incidence for platelets and neutrophils was compared using Gray’s test. The Wilcoxon rank-sum test was used to compare immune recovery parameters at the different time intervals. Since the distributions of the cytokine levels are skewed to the right with extremely large values, we performed a logarithm transformation on these variables before analysis to make the distributions close to normal. Then, the cytokine levels were compared using the Wilcoxon rank-sum test between the two groups, and univariate linear regression models were fit to correlate times to ANC and platelet recovery with each of the (log) cytokine levels. Kaplan-Meier estimates of the survival curves for time to relapse, progression-free survival, and overall survival were obtained, and the log-rank test was used to compare survival curves. A logistic regression model with ordinal responses was fitted to compare the occurrence of graft-versus-host disease (GVHD) between the two groups.

## Results

### Patient characteristics

Thirty-one patients consented to participate in the trial. One patient withdrew consent before receiving study drug. Thirty patients were eligible for analysis. The baseline characteristics of the patients are provided in Table 1. The study cohort and comparator cohort were well balanced with respect to age, disease, disease status, donor type, and graft source. The majority of patients in the plerixafor cohort received either TBI\cyclophosphamide (40 %) or busulfan\cyclophosphamide (43 %) conditioning while in the other group, the main preparatory regimen was busulfan and cyclophosphamide (35 %). Sixty-three percent of the patients in the plerixafor group had matched unrelated donors. Ninety-four percent of the patients in plerixafor group received peripheral blood stem cells as their graft source, and 6 % of the patients (two patients) in plerixafor group received BM graft. All of the patients in the historical control cohort had peripheral blood stem cells as their graft source.

### Adverse effects

Plerixafor was well tolerated with no grade 3 or higher adverse events that could be directly attributed to plerixafor. The phase I portion did not lead to development of any plerixafor-related toxicities, and therefore, the phase II portion was initiated. There were no dose-limiting toxicities of premature ventricular arrhythmias, primary or secondary graft failure, or mortality directly associated with plerixafor administration. The most common grade 1 or 2 adverse events detected were gastrointestinal, such as abdominal pain, bloating, diarrhea, and nausea. These were difficult to distinguish from effects of the myeloablative conditioning. There was one patient with grade 2 diarrhea that was attributed to plerixafor administration. One patient treated with plerixafor developed atrial fibrillation.

### Engraftment kinetics

#### Neutrophil recovery

**Fig. 2:**
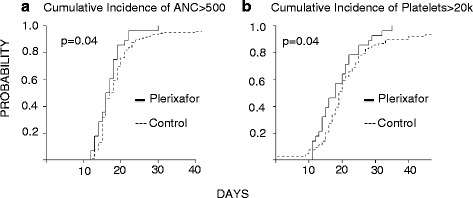
Cumulative incidence of neutrophil engraftment and platelet engraftment. *Left panel*: cumulative incidence of neutrophil engraftment in plerixafor-treated patients (*solid line*) and control patients (*dashed line*). *Right panel*: cumulative incidence of platelet engraftment in plerixafor-treated patients (*solid line*) and contemporaneous control patients (*dashed line*). Excluding the two bone marrow recipients

The median time to neutrophil recovery was 17 days for both the study cohort and the control cohort (*p* = 0.22). Comparison of the cumulative incidence curves for neutrophil recovery suggests an enhancement in ANC recovery for the plerixafor cohort; however, this difference was not statistically significant (*p* = 0.07) when all 30 plerixafor-treated patients including the two BM graft recipients were included for analysis. If the two plerixafor patients receiving BM graft were removed and only patients receiving peripheral blood stem cells were compared, the difference in the cumulative incidence of neutrophil recovery between plerixafor cohort and the control group was statistically significant (*p* = 0.04) (Fig. 2a).

#### Platelet recovery

The median time to platelet recovery was 18 days for the plerixafor study group and 19 days for the control cohort. Comparison of the cumulative incidence curves demonstrates more rapid and enhanced platelet recovery for those receiving post-transplant plerixafor either when all 30 patients or only the 28 peripheral blood stem cell (PBSC) recipient patients were included for analysis (*p* = 0.04) (Fig. 2b).

### Acute graft-versus-host disease

Of the 30 patients who completed the study, 17 patients (57 %) experienced none or grade I acute GVH compared to 57 % in the contemporaneous control (*p* = 1). Grade II–IV acute GVH was 43 % in both groups (*p* = 0.6). Grade III–IV acute GVH was 23 % in the comparator control cohort compared to 16 % in the plerixafor treatment group (*p* = 0.62) (Table 2).

**Table 2:** Occurrence of acute GVHD

Category	Plerixafor group	Control group	*p* value
	*n* = 30	*n* = 14	0.90
Acute GVHD			
None or grade I	17 (56 %)	54 (57 %)	1.00
Grade II	8 (26 %)	19 (20 %)	0.60
Grades III–IV	5 (16 %)	22 (23 %)	0.62

*Note*: Comparisons among the two groups were performed by the use of the extended Fisher exact test for acute GVHD

### Survival

Of the 30 patients who underwent treatment, two patients died before day 100. Transplant-related mortality calculated at day +100 with 95 % CI was 0.069 (0.00, 0.157). Progression-free and overall survival were comparable between plerixafor-treated patients and contemporaneous controls (progression-free survival (PFS) *p* = 0.68, overall survival (OS) *p* = 0.97). Day 100 and 1 year PFS were 78 and 60 % in the plerixafor-treated patients. The control cohort had calculated day 100 and 1 year PFS of 93 and 42 %, respectively. OS for the plerixafor group compared to the untreated group at day 100 and 1 year are (day 100) 85 % (plerixafor) versus 93 % (comparator group) and (1-year) 65 % (plerixafor) and 57 % (comparator). Disease relapse rates were also similar between the two groups (*p* = 0.87). The study group had a day 100 relapse rate of 12 % compared with 0 % in the control cohort. One-year relapse rates were 23 and 34 %, respectively, between the study group and the comparator cohort, while 2-year relapse rates were 26 % (plerixafor) and 34 % (comparator) for the two groups.

### Immune reconstitution

Overall, there were no observable trends to suggest a difference in the kinetics of immune recovery between the treatment and control group. The absolute lymphocyte count at day +90 was significantly higher in the control group (median 1010) compared to the treatment group (median 549; *p* = 0.02). Similarly, in the day +90, CD3+ cell count was higher in the control group (median 655) than the treatment group (median 409) (*p* = 0.04). Other than these findings, there were no statistically significant differences between pre-transplant, day +30, day +60, and day +90 immune recovery kinetics in absolute lymphocyte count, NK cell count, CD3+, CD4+, CD8+, NKT cells, plasmacytoid dendritic cells, and myeloid dendritic cells.

### Inflammatory cytokine measurements

Correlative studies were performed to analyze differences in inflammatory cytokines in patient samples at varying time points. Plasma samples were collected from all plerixafor-treated patients at day +7, day +14, and day +30 and were assayed for IFN-γ, TNF-α, IL-12, and MIP-1β. Plasma samples collected at day +14 and day +30 from 20 of the control patients were used for comparison. Eleven plerixafor-treated patients had BM biopsy at day 30, and the BM supernatants were prepared and assayed for the four cytokines.

**Fig. 3:**
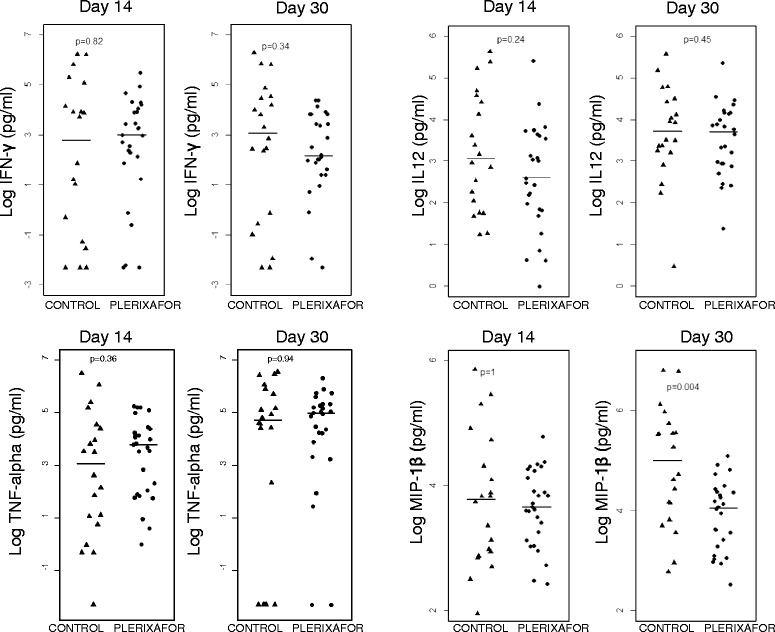
Plasma cytokine levels measured at day 14 and day 30

Compared to blood plasma samples, BM microenvironment tended to have a lower level of IFN-γ, TNF-α, IL-12, and MIP-1β (data not shown). There were no statistically significant differences in plasma IFN-γ, TNF-α, IL-12, and MIP-1β levels between plerixafor-treated patients and controls at day 14 (data not shown). Interestingly, the plasma MIP-1β level was significantly lower at day +30 in plerixafor-treated patients (61.3 ± 40.1 pg/ml, mean ± SD) compared to the control (237.0 ± 257.8 pg/ml) (*p* = 0.004). The levels of plasma IFN-γ, TNF-α, and IL-12 were comparable between plerixafor-treated patients and controls at day +30 (Fig. 3).

**Fig. 4:**
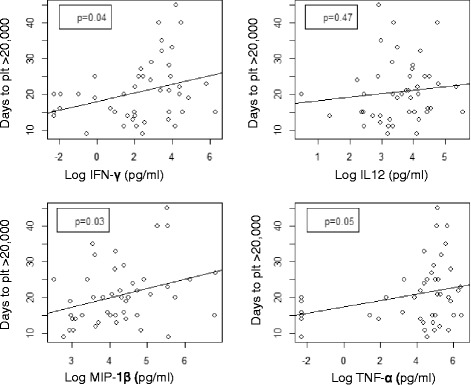
Correlation analysis between the cytokine level at day 30 and the days of platelet engraftment

Analyses were performed to determine the correlation between the plasma cytokine levels and the time to neutrophil recovery or platelet recovery (Fig. 4). The cytokine levels at either day 14 or day 30 did not correlate the speed for neutrophil recovery. Neither did the cytokine levels measured at day 14 correlate with the days of platelet engraftment (data not shown). Interestingly, however, the levels of plasma MIP-1β and IFN-γ at day 30 inversely correlated with platelet recovery (*p* = 0.03 and 0.04, respectively), that is, the higher the MIP-1β or IFN-γ level, the longer it takes for platelet engraftment (Fig. 4). There appears to be a trend for similar correlation for TNF-α measured at day +30 post-HSCT (*p* = 0.05) (Fig. 4).

## Discussion

We performed a phase I/II clinical study aimed at determining the safety and efficacy of plerixafor (a specific and reversible CXCR4 antagonist) after allogeneic HSCT to enhance hematopoietic cell recovery in patients receiving myeloablative allogeneic HSCT. Plerixafor was extensively explored as a stem cell mobilizer and approved by the FDA to be used in the pre-transplant setting for stem cell collection [12–15]. Contradictory to the dogma related to CXCR4 blockade, we previously found that post-transplant administration of plerixafor led to enhanced recovery of donor-derived cells in a murine transplant model [11], and our results were confirmed by others using SDF-1 conditional knockout mice [16]. In the current study, we demonstrate that patients treated with plerixafor have a shorter time to platelet recovery compared to contemporaneous controls (18 versus 19 days) and a higher cumulative incidence of platelet recovery compared to the control cohort. In addition, there is a trend toward enhanced neutrophil recovery in plerixafor-treated patients (*p* = 0.07). Unplanned subgroup analyses in fact demonstrate a statistically significant difference when the two study patients who received BM grafts are removed (*p* = 0.04). Our preclinical and clinical data demonstrates that blockade of the CXCR4-SDF1 axis alters the BM niche and promotes stem cell proliferation [11, 17]. This is the first study reporting the beneficial effects of post-transplant administration of plerixafor in enhancing donor cell engraftment following myeloablative allogeneic HSCT.

Adverse events attributable to plerixafor were modest, expected (GI upset, nausea, diarrhea, fatigue) and difficult to distinguish from effects of the myeloablative conditioning. There were no dose-limiting toxicities of primary or secondary graft failure or mortality associated with plerixafor administration. Taken together, this study provides evidence that administration of plerixafor following allogeneic HSCT is safe and feasible.

Post-transplant administration of plerixafor enhances hematologic recovery via several mechanisms. We have previously shown that giving plerixafor after HSCT mobilized residual recipient cells into circulation and increased niche availability for donor HSCs to engraft [11]. Furthermore, we found that post-transplant administration of plerixafor induced HSC cell division in vivo [11]. CXCR4/SDF-1 interaction has two major functions: tethering HSCs in niches and maintaining HSCs in a quiescent state. However, in HSCT, rapid expansion and proliferation of HSCs are crucial and desired especially during the early stage of hematological recovery [18, 19]. Plerixafor has a terminal half-life of 5.3 h [20]. Thus, giving plerixafor once every other day results in transient and pulsed blockade of SDF-1/CXCR4, inducing HSC expansion and proliferation without the risk of HSC exhaustion [11, 16].

Transplant preparatory conditioning induces massive cell death and tissue damage, resulting in a dramatic increase in the levels of secreted chemokines, cytokines, and proteolytic enzymes [21]. These cytokines/chemokines in turn further exacerbate inflammation and vascular/tissue injury, leading to multi-organ toxicity (cytokine storm). Additionally, several of these cytokines/chemokines including TNF [8–10], TGF-β [22], IL-3 [23], and IFN-γ [7] have negative effects on hematopoiesis. In mouse models, we previously found that post-transplant administration of plerixafor led to a significant reduction in the levels of nine pro-inflammatory cytokines/chemokines [11]. Additionally, we demonstrated that in the early phase of transplant, the marrow microenvironment of transplant recipient mice contains soluble factors that inhibit hematopoiesis and that post-transplant administration of plerixafor likely reduces these inhibitors and/or increases factors that promote hematopoiesis [17]. Consistent with our preclinical studies, our correlative inflammatory cytokine studies demonstrated a reduction in MIP-1β expression at day 30 when compared to patient samples from the historical control cohort. MIP-1β is an inflammatory cytokine with various affects under physiologic conditions. Our current studies are limited to the measurement of only four cytokines (IL-12, TNF-α, IFN-γ, and MIP-1β). These four cytokines were selected based on our preclinical studies and are important mediators in inflammatory process. It would be important to expand the measurement to many other cytokines. Nevertheless, our finding supports the role of CXCR4 antagonism in attenuating inflammatory cytokine storm following allogeneic HSCT.

Compared to what was observed in our preclinical mouse studies, the augmentation of engraftment kinetics by post-transplant plerixafor observed in this clinical trial was modest. There are several potential reasons for this. In our animal study, plerixafor was given at 5 mg/kg body weight whereas in our clinical trial, plerixafor was given at 240 μg/kg body weight. Second, in our animal study, plerixafor was given every other day until day +56 post-HSCT whereas in our clinical trial, plerixafor was administrated every other day until neutrophil engraftment, which came at a median of 16 days following transplantation. This may have dampened the beneficial effect of plerixafor on platelet recovery. Third, in our animal model, the recipient mice were transplanted with a small dose of donor stem cells (250 stem cells per mouse). In contrast, all our patients received on average 5 × 10^6^ CD34 cells/kg (ranging from 1.25 × 10^6^ to 8.5 × 10^6^ CD34 cells/kg). Such a high dose of donor stem cells likely masked the beneficial effects of CXCR4 blockade on stem cell engraftment and proliferation. Finally, although we chose contemporaneous control patients for comparison, there are differences between these two groups of patients. For instance, 36 % of plerixafor-treated patients were ALL patients versus 11 % in control cohort. Forty percent of plerixafor-treated patients received conditioning with TBI + cyclophosphamide compared to only 21 % in control cohort. Furthermore, because of the small patient size, our analysis was not adjusted for CD34 cell dose or ideal body weight. These variables could potentially impact the effects of plerixafor seen in this clinical study.

Our preclinical studies demonstrated an improvement in immune reconstitution among mice treated with plerixafor compared to those given PBS. This finding was not observed in this study. This may be secondary to a multitude of factors including confounding by immunosuppressive medications, clinical status at the time of blood draw, and other homeostatic differences between individual patients.

Currently, recombinant G-CSF is the only agent approved by FDA for enhancing neutrophil recovery following HSCT. G-CSF, however, impacts only myeloid recovery and improves the time to neutrophil recovery following HSCT by only a few days [24, 25]. There is an unmet medical need to identify agents that improve the kinetics of recovery of multiply hematopoietic lineages. This study suggests that plerixafor may be such an agent. It is also noteworthy that plerixafor does not appear to negatively impact OS or disease relapse: the 1-year OS in plerixafor-treated patients was 65 % compared to 57 % in control group, and the 2-year disease relapse rate was 26 % in plerixafor-treated patients versus 34 % in control cohort. We believe that our study has potentially important ramifications in improving hematopoietic recovery in myeloablative HSCT.

Our study provides the foundation for the efficacy of manipulating the CXCR4-SDF1 interaction to provide clinical benefit to recipients of HSCT. Moving forward, we plan to study plerixafor administration following umbilical cord blood transplantation, where the stem cell dose is lower and hematopoietic recovery is significantly slower. Second, a large study controlled for CD34+ cell dose, ideal body weight, and disease characteristics will help further determine the beneficial effects of plerixafor in enhancing hematopoietic recovery. Third, plerixafor has a short half-life (a few hours), and in this study, it was given every other day. Therefore, more frequent dosing higher dose more consistent with what was used in the preclinical studies will be explored. Finally, other CXCR4-SDF1 antagonists with greater affinity to the site of action and with longer half-lives may result in a more clinically meaningful effect and deserve further study.

## Conclusions

In summary, blockade of the CXCR4-SDF1 axis post-allogeneic HSCT is safe. While modest, it enhances hematopoietic recovery in patients who have received matched HLA transplants. Further confirmatory studies are justified, potentially in populations with anticipated delays in hematopoietic recovery. Newer CXCR4 antagonists are in varying stages of development and may have utility in this setting in the future.

## Abbreviations

HSCT: Hematopoietic stem cell transplantation
HSPC: Hematopoietic stem/progenitor cell
PBSC: Peripheral blood stem cell
BM: Bone marrow
SDF-1: Stromal-derived factor-1
GvHD: Graft-versus-host disease
HLA: Human leukocyte antigen
TBI: Total body irradiation
ANC: Absolute neutrophil count
OS: Overall survival
PFS: Progression-free survival
